# Fitness consequences of *Anopheles gambiae *population hybridization

**DOI:** 10.1186/1475-2875-4-44

**Published:** 2005-09-20

**Authors:** David M Menge, Tom Guda, Daibin Zhong, Aditi Pai, Goufa Zhou, John C Beier, Louis Gouagna, Guiyun Yan

**Affiliations:** 1 Department of Biological Sciences, State University of New York at Buffalo, Buffalo, NY 14260, USA; 2 Mbita Point Field and Training Station, International Center of Insect Physiology and Ecology, P.O. Box 30772, Nairobi, Kenya; 3 Department of Epidemiology and Public Health, University of Miami, Miami, FL 33177, USA; 4 Institut de Recherche pour le Développement (IRD) UR 016/ LIN, 911 avenue Agropolis, BP 64501 34394 Montpellier Cedex 5, France

## Abstract

**Background:**

The use of transgenic mosquitoes with parasite inhibiting genes has been proposed as an integral strategy to control malaria transmission. However, release of exotic transgenic mosquitoes will bring in novel alleles along with parasite-inhibiting genes that may have unknown effects on native populations. Thus it is necessary to study the effects and dynamics of fitness traits in native mosquito populations in response to the introduction of novel genes. This study was designed to evaluate the dynamics of fitness traits in a simulation of introduction of novel alleles under laboratory conditions using two strains of *Anopheles gambiae*: Mbita strain from western Kenya and Ifakara strain from Tanzania.

**Methods:**

The dynamics of fitness traits were evaluated under laboratory conditions using the two *An. gambiae *strains. These two geographically different strains were cross-bred and monitored for 20 generations to score fecundity, body size, blood-meal size, larval survival, and adult longevity, all of which are important determinants of the vector's potential in malaria transmission. Traits were analysed using pair-wise analysis of variance (ANOVA) for fecundity, body size, and blood-meal size while survival analysis was performed for larval survival and adult longevity.

**Results:**

Fecundity and body size were significantly higher in the progeny up to the 20^th ^generation compared to founder strains. Adult longevity had a significantly higher mean up to the 10^th ^generation and average blood-meal size was significantly larger up to the 5^th ^generation, indicating that hybrids fitness is enhanced over that of the founder strains.

**Conclusion:**

Hybridization of the two mosquito populations used in this study led to increased performance in the fitness traits studied. Given that the studied traits are important determinants of the vector's potential to transmit malaria, these results suggest the need to release genetically modified mosquitoes that have the same or very similar backgrounds to the native populations.

## Background

Malaria is one of the most fatal infectious diseases in the tropics despite continued efforts to contain it [1-3]. Manipulating vector competence to lower transmission efficiency has been proposed as a possible integral component in the control of malaria transmission [4-6]. It is expected that manipulation, such as the introduction of fertile mosquitoes transformed with anti-parasite molecules, will result in a population of mosquitoes with compromised biological ability to facilitate development and transmission of the malaria parasites [5]. Over the past several years, remarkable progress has been made in mosquito germ-line transformation and in the identification of parasite-inhibiting molecules [5,7] that can be incorporated into a genetically modified vector to render it incapable of transmitting the malaria parasite. For example, *Anopheles gambiae *cell lines have been successfully transformed with the *Hermes *element [8,9] and the *Minos *transposable element, bearing an exogenous gene, has been efficiently integrated into the genome of *Anopheles stephensi *[10]. A number of genes inhibiting malaria parasite development in the mosquito vectors have been identified, including single-chain monoclonal antibodies [11], salivary-midgut peptide [12], phospholipase A2 [13] and mosquito innate immune genes [14,15]. It has been shown that genetically-modified mosquitoes with parasite-inhibiting genes can efficiently suppress parasite infection intensity in mosquitoes in the laboratory [5,14]. The availability of the complete genome sequence of *An. gambiae *provides an unprecedented opportunity to study the genetic aspects of malaria transmission and to identify new targets for transmission blocking [17].

The success of the transgenic mosquito approach will depend on the spread and even the fixation of parasite-inhibiting genes into natural vector populations [4,6]. Despite the progress in mosquito genetic transformation and the isolation of parasite-inhibiting genes, little has been done to determine how the fertile genetically-transformed mosquitoes should be released or how fast the introduced genes will spread in natural populations. Previous studies suggest that 'gene-driving' mechanisms are needed to accelerate the spread and fixation of introduced genes in natural populations of competent vectors [18-20]. These genes can be driven to fixation either using transposable elements or through Wolbachia-induced cytoplasmic incompatibility [21]. However, no matter which gene-driving mechanism is used, releasing transgenic mosquitoes in nature will require careful laboratory work and controlled field studies to determine the rate of gene-spread and to assess biosafety issues [19]. Thus, before any transgenic mosquitoes are released to the environment, the dynamics of released genes should be well understood. The mosquitoes to be released are likely to be transgenes of recent anopheline colonies from native populations; alternatively, exotic transgenic mosquitoes may be released. In either scenario, novel alleles in addition to parasite-inhibiting genes may be introduced to the environment, and it is necessary to evaluate and understand the dynamics of these novel alleles. The fitness and feeding behaviours of hybrids between the introduced and native mosquito populations should be evaluated since these hybrids may become an increased nuisance, if they bite humans more vigorously or survive longer than native mosquitoes.

This paper reports the results of an experiment designed to evaluate the dynamics of genes under laboratory conditions using *An. gambiae *Mbita strain from western Kenya and Ifakara strain from Tanzania. These two strains were crossbred and monitored up to the 20^th ^generation to determine: a) if the dynamics of gene frequency from either strain can be predicted based on selected fitness traits and b) at what generation the fitness traits stabilize. Since the two founder populations are geographically different, it is likely that the hybrids' fitness will be enhanced in the initial crosses. The fitness traits studied were blood-meal size, adult body size (measured by wing length), fecundity, larval survivorship, and adult longevity. These traits are important indicators of vector fitness as discussed in Yan et al [18].

## Methods

### Mosquito rearing and maintenance

Two strains (Mbita and Ifakara) of laboratory-reared *An. gambiae *mosquitoes were used in this study. The Mbita strain was originally collected at Mbita Point (000 25'S, 340 13'E) in west Kenya and has been maintained in the laboratory since 1999. The Ifakara strain was originally collected in Njage village, 70 km from Ifakara, southeastern Tanzania, and has been maintained under laboratory conditions since 1996. The colonized populations did not exhibit a significant reduction in the observed heterozygosity in six microsatellite markers, including AGXH1D1 and AGXH131 of chromosome X, AG2H46 and AG2H79 of chromosome 2, and AG3H29C and AG3H33C of chromosome 3 (D. Zhong and G. Yan, unpublished data).

The populations of the two parent strains used in this study were raised from mosquito eggs obtained from the existing colonies maintained at the International Centre for Insect Physiology and Ecology (ICIPE), Mbita Point Field Station in the mosquito-rearing insectary. Post- mated females were allowed to engorge from a volunteer's arm for 15 minutes to facilitate oviposition. Fully engorged females were isolated from non-engorged ones and placed in single oviposition cups. Each oviposition cup contained a wet Whatman filter paper disc at the bottom and a strip of the same Whatman filter paper leaning against the walls of the oviposition cup for the mosquito to perch on. During the oviposition period, the mosquitoes were maintained on a diet of glucose solution (6%) provided by a wick of cotton placed on top of the mesh covering the oviposition cup.

The oviposited eggs were collected from the wet filter paper disks in the oviposition cups and transferred to plastic containers of distilled water. Upon hatching, the first in-star larvae were transferred to plastic trays measuring 21 cm in diameter. Larval densities were maintained at 300 larvae per tray in distilled water maintained at a depth of 8 cm. Trays containing larvae were fed daily on 30 mg of Tetramin^® ^fish food and were subjected to natural ambient light. On pupation, the pupae were moved to standard 30 × 30 × 30 cm netting cages. After emergence, adult mosquitoes were held in cages and were offered 6% glucose and distilled water by means of moistened cotton wick placed on top of the mesh on the cages. The cages were kept in the insectary at ambient tropical temperature and under artificial light provided by fluorescent tubes. Relative humidity was maintained at ambient by basins of water placed in the insectary.

Hybrids of the F_1 _generation from the two strains were obtained mixing 100 Ifakara females, 100 Ifakara males, 100 Mbita females, and 100 Mbita males in a single 30 × 30 × 30 cm cage. The F_1 _hybrid larvae were reared to adults under similar conditions. Subsequent generations from F_2 _to F_20 _were obtained by mass mating and reared under similar conditions with a population size maintained at approximately 1,000 individuals.

### Measurement of fitness components

The fitness of mosquitoes sampled from cage populations of the two single strains, Mbita and Ifakara (F_0_), and the hybrids between the two strains at the F_1_, F_5_, F_10_, F_15_, and F_20 _generations, was measured. In order to confirm the direction and effect of heterosis and also to control any assortative mating of mixed populations, reciprocal matings of Mbita male and Ifakara female and vice versa were was carried out, and their F_1 _and F_5 _progeny were subjected to measurement of fitness traits. The fitness components studied included mosquito fecundity, body size (measured by wing length), blood-meal size, larval survival, and adult longevity. All of these fitness traits were measured in two replicate experiments. Because the traits measured in this study could be sensitive to environmental parameters, the populations and filial generations used were reared in the same manner as previously described. The differences observed among the two founder populations and their different filial generations are therefore likely to be due to their genetic differences.

#### (i) Fecundity

Fecundity is an important fitness trait as it indicates the efficiency of conversion of the blood meal to eggs and it also influences the number of offspring from a single female mosquito that are likely to survive to become adults [22,23]. Fecundity was scored as the number of eggs laid plus any eggs retained in a single gonotrophic cycle. Females were fed as previously described, and gravid females (n = 50 in two replicates) were then transferred to individual glass tubes for oviposition. The number of eggs laid by each individual was recorded. Any female who died before or after oviposition was dissected, and any retained eggs were counted. In most cases, no eggs were retained. When eggs were retained, those eggs contributed less than 1% of the total number of eggs in that gonotrophic cycle.

#### (ii) Wing length

Wing length is a reliable correlate of mosquito body size [22,23]. As a fitness trait, mosquito size may influence survivorship, developmental time, and the ability to acquire a blood meal; it also has a positive correlation to fecundity [23]. Wing length was measured from the same individuals (n = 50 for two replicates) used to measure fecundity. One wing was removed at the time of dissection, and wing length was measured from the axial incision of the apical margin as described by Kelly and Edman [23]. Each wing was mounted on a glass microscope slide in a small drop of distilled water. Wing length was measured to the nearest 0.01 mm using a compound microscope.

#### (iii) Blood-meal size

Blood-meal size influences both mosquito fecundity and the potential to acquire pathogens [24,25]. Indirect quantification of blood-meal size was conducted based on the principle of haemoglobinometry using the HiCN method [26]. Starved three-day-old females were allowed to feed from a volunteer's arm for 15 minutes. Blood-fed females (n = 50 for two replicates) were immediately frozen and thoroughly ground in individual tubes; Drabkins reagent was then added. This reagent converts all haemoglobin (Hb) to a cyano-derivative whose color intensity is proportional to total Hb concentration in the blood-fed mosquito. This mixture was then vortexed and centrifuged at 1,500 rpm for five minutes. The supernatant was transferred to a test tube with 800 μl distilled water and vortexed briefly. The absorbance was read at 415 nm using a Chemlab Spectronic 20D spectrophotometer.

A standard curve was prepared for each of the assays done on the Mbita, the Ifakara, and their filial generations using known volumes of human blood (0.8, 1.6, 2.4, 3.2, and 4.0 μl) and measuring the corresponding absorbance. The absorbance of unfed female mosquitoes (n = 5) was also measured, and the average of their absorbance reading was used as a correction factor for the absorbance obtained from blood-fed females.

#### (iv) Larval and adult survivorship

Larval survivorship has a significant influence on the number of pupae and adult mosquitoes that emerge. Adult longevity, on the other hand, influences the number of gonotrophic cycles and the potential for pathogen transmission. For determining larval survivorship, 50 first in-star larvae of the founder populations and their filial generations were transferred into distilled water maintained at a depth of 8 cm in plastic trays measuring 21 cm in diameter. Trays containing larvae were fed daily with 30 mg of Tetramin^® ^fish food, and were subjected to natural ambient light, and monitored daily. Dead larvae were recorded and removed; larvae that pupated were also recorded, removed, and then transferred to emergence cages. Mosquito adult survivorship of the two founder populations and their respective filial generations were examined. In each case, 50 individuals were put in a cage in the insectary and maintained on a glucose diet, as previously described. Mosquitoes were examined daily and dead individuals were counted and removed until the day the last individual died.

### Data analysis

The original fitness traits data were analysed using analysis of variance (ANOVA) for fecundity, body size, and blood-meal size. Larval and adult survival were subjected to survival analysis to compare the performance of phenotype traits between the founder parents, reciprocal crosses and each filial generation. Comparisons were also made between successive filial generations to infer the level at which maximum genotype hybridization was achieved. The significance of all statistical tests was set at 0.05. Statistical analyses were performed in MINITAB (Minitab Inc.) and SPSS (SPSS Inc.) computer software. The dynamics of population hybridization were also simulated in random-mating generations founded by Mbita strain (A) and Ifakara strain (B), using p(AB_t+1_) = 1 - p(A_t+1_) - p(B_t+1_), where p(AB_t+1_) is the proportion of hybrids between the two strains at generation t, p(A_t+1_) is the proportion of Mbita homozygotes at generation t, and p(B_t+1_) is the proportion of Ifakara homozygotes at generation t.

## Results

### Fecundity

There was a significant difference in fecundity (p < 0.001) between the Mbita and Ifakara strains, with the Ifakara strain having at least 20% more eggs than the Mbita strain in the two replicate measurements (Table 1). There was also a significant difference (p < 0.001) in fecundity between the parental strains and their hybrid progeny at F_1_, F_5_, F_10_, F_15_, and F_20 _in the two replicates studied in this experiment. The hybrid progeny had a higher mean, suggesting a high degree of heterosis for this trait. A pair-wise analysis of variance between F_1 _and F_5_, F_5 _and F_10_, F_10 _and F_15_, and F_15 _and F_20 _showed no significant difference in fecundity (results not shown). There was a significant difference in the fecundity of F_1 _and F_10 _(p < 0.05), but no significant difference between F_1 _and F_15 _or F_15 _and F_20 _(results not shown). Likewise, there was no significant difference in fecundity between F_5 _and F_20 _or F_10 _and F_20_.

**Table 1:** Summary of fitness traits for Mbita and Ifakara strains, and filial generations of hybridization between the two strains (100 Ifakara females, 100 Ifakara males, 100 Mbita females, and 100 Mbita males).

Trait	Replicate	Mbita	Ifakara	F_1_	F_5_	F_10_	F_15_	F_20_
Wing length (mm)	1	2.92 (0.02)	2.96 (0.02)	3.10 (0.02)	3.09 (0.03)	3.11(0.02)	3.14(0.03)	3.03(0.16)
	2	2.87 (0.03)	2.89 (0.03)	3.17 (0.03)	3.34 (0.03)	2.98(0.02)	3.07(0.03)	2.95(0.02)
Fecundity*	1	47.17(2.66)	58.25(2.29)	63.37(2.28)	64.58(2.18)	70.26(2.05)	65.08(2.02)	68.90 (1.83)
	2	35.96(2.56)	49.28(2.67)	62.60(2.95)	65.42(2.32)	76.97(2.80)	63.60(2.23)	61.44(2.38)
Blood-meal size (μl)	1	3.15(0.14)	2.54(0.08)	3.96(0.26)	2.78(0.15)	2.53(0.23)	3.10(0.15)	3.13(0.16)
	2	2.93 (0.18)	2.61 (0.11)	4.51 (0.21)	4.02 (0.16)	2.97 (0.20)	3.19 (0.17)	3.21 (0.13)
Larval survivorship (%)	1	91.78	94.44	98.00	95.22	97.00	95.78	95.88
	2	96.44	92.54	98.34	93.34	96.66	93.44	95.78
Mean adult longevity (days)	1	22.31	14.52	20.03	29.00	31.81	20.71	16.95
	2	19.61	15.66	19.97	21.66	33.72	16.38	18.64

Note: The values in parenthesis are standard errors. * Fecundity is the number of eggs laid plus the number of eggs retained.

### Wing length

No significant difference in wing length was found between the Mbita and Ifakara strains (Table 1). However, there was a consistent significant difference between the two founder strains and their F_1_, F_5_, F_10_, F_15_, and F_20 _progeny in the two replicates (p < 0.05). This can be attributed to heterosis for this trait. The inter-progeny difference was significant between F_1 _and F_5 _(p < 0.05) but not between F_5 _and F_10_, F_10 _and F_15_, and F_15 _and F_20_(results not shown).

### Blood-meal size

An analysis of variance showed a significant difference in blood-meal size between the Mbita and Ifakara strains (Table 1; p < 0.001) with the Mbita strain taking a larger blood meal. There was also a significant difference in blood-meal size between the founder populations – Mbita and Ifakara – and the F_1 _progeny (p < 0.05). All of the other filial generations had a significantly larger blood-meal size than the Ifakara strain (p < 0.001), whereas the F_5_, F_10_, F_15_, and F_20 _generations' blood-meal size did not differ significantly from the Mbita strain (Table 1). A pair-wise analysis of variance showed significant difference between F_1 _and F_5 _(p < 0.001), but not between F_5 _and F_10_, F_10 _and F_15_, and F_15 _and F_20_. Mean blood-meal size showed significant difference between F_1 _and F_10_, F_1 _and F_15_, and F_1 _and F_20_, whereas there was no significant difference between the means of F_5 _and F_15_, and F_5 _and F_20_. The mean blood-meal sizes of F_10 _and F_20 _were also not significantly different.

### Larval survivorship and adult longevity

Adult longevity was significantly greater in the Mbita strain compared to the Ifakara strain. The mean longevity of the filial generations showed an increasing trend from F_1_, F_5_, and F_10, _which had the greatest longevity due to heterosis (Table 1). From F_10_, the mean longevity showed a decreasing trend in F_15 _and F_20 _(Table 1). Data for larval survival did not show significant consistent differences between the founder parents and the filial generations – unlike with the other traits – because more than 90% of the larvae in all experiments survived to become pupae (Table 1).

### Reciprocal crosses

Data on reciprocal crosses of males and females of either Mbita or Ifakara strains showed significantly higher means of body size, fecundity, and blood-meal size at both the F_1 _and F_5 _generations than either founder strain (Table 2). This confirms that enhanced fitness in the progeny of Mbita and Ifakara strains is due to heterosis between the two genotypes.

**Table 2:** Means of fitness traits for Mbita and Ifakara strains and their respective female/male crosses

Strain/Population	Wing length in mm (standard error)	Fecundity* (standard error)	Blood-meal size (μl) (standard error)
Mbita	2.86 (0.03)	35.97 (2.56)	3.15 (0.14)
Ifakara	2.89 (0.03)	49.27 (2.67)	2.54 (0.08)
F_1 _from Mbita female × Ifakara male	3.13 (0.03)	74.87 (2.63)	4.04 (0.16)
F_1 _from Ifakara female × Mbita male	3.03 (0.02)	83.20 (5.36)	4.51 (0.21)
F_5 _from Mbita female × Ifakara male	2.94 (0.02)	68.93 (2.61)	4.02 (0.16)
F_5 _from Ifakara female × Mbita male	3.14 (0.02)	68.77 2.69)	3.38 (0.18)

* Fecundity is the number of eggs laid plus the number of eggs retained.

### Population hybridization

**Figure 1:**
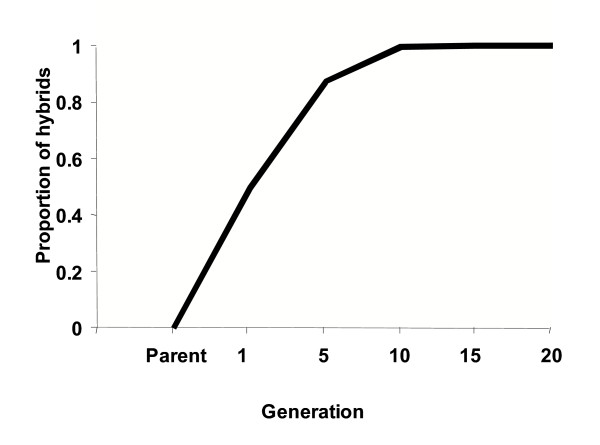
**Plot of expected proportion of hybrids against time (Generation). **The simulation assumed equal sex ratio and equal number of *Anopheles gambiae *Mbita and Ifakara strains in random mating, as in the experimental set up.

Computer simulation indicates the proportion of hybrids between Mbita and Ifakara strains increases continuously and reaches to 100% after 10 generations (Figure 1).

## Discussion

In this study, the fitness consequences of population hybridization were examined in 20 filial generations from crosses between two geographically different *An. gambiae *strains. Four fitness components were examined: fecundity, body size, blood-meal size and adult survival. These fitness traits are important determinants of the vector's potential to transmit the malaria parasites (*Plasmodium *species) and should, therefore, be considered in strategies aimed at controlling malaria transmission through the introduction of exotic and genetically modified mosquitoes. The results of this study show that fitness traits are subject to the effects of heterosis, i.e., hybrids have increased values for fitness traits than do the parent populations. Mosquito body size (measured as wing length) and fecundity showed heterosis up to the 20^th ^generation. In both replicates, the 20^th ^generation had significantly higher values (at least 5% longer wing length and 20% more eggs) than either parent. Blood-meal size also showed heterosis, with the F_1 _and F_5 _generations imbibing at least 50% more blood than the Ifakara strain. Subsequent generations were not significantly different from the Mbita strain, but showed significantly greater blood-meal size than the Ifakara strain. Adult longevity showed equally robust heterosis in the hybrid generations. The F_1_, F_5_, and F_10 _generations had, respectively, 40%, 66% and 100% higher mean longevity than the founding Ikafara strain; the 15^th ^and 20^th ^generations had mean longevity that was between the two founder strains but was higher than the Ifakara strain. Further analysis indicated that the maximum heterosis for body size, fecundity, and adult longevity occurred at the 10^th ^generation, after which there was no significant pair-wise difference.

These results can be attributed to increasing hybridization of the Ifakara and Mbita strains. In the experimental design used in this study, 100 adult mosquitoes of each sex from the two strains were put together to mate. In the first generation, there were more (Mbita × Mbita) and (Ifakara × Ifakara) matings. In subsequent generations, the genotypes mating were Mbita, Ifakara, and intercrosses of the founder populations, and therefore the probability of within-strain mating (Mbita × Mbita; or Ifakara × Ifakara) reduced from generation to generation. The increasing trends of heterosis in the studied traits were due to increasing hybridization of the Mbita and Ifakara strains, as demonstrated in Figure 1. On attaining maximum hybridization, heterosis is maximally expressed, as reflected by the performance of the fitness traits. As would be expected [27], this is then followed by eventual decline in performance due to the waning effect of heterosis.

## Conclusion

The results of this study show that hybridisation of different *Anopheles gambiae *populations lead to enhanced fitness than the parental populations. In the proposed use of transgenic mosquitoes as a strategy to control malaria transmission, it is expected that transgenic mosquitoes with the inherent ability to resist malaria parasite development will be released into the field [4-6]. By cross-mating with native wild populations, transgenes are expected to spread out and be fixed in the native wild population to render it incapable of supporting parasite development and transmission [20]. If the transgenic mosquitoes are exotic to the region, the possibility exists that hybrids will have greater fitness than native mosquitoes. As shown by the experiments done in this study, the progeny of the Mbita and Ifakara strains have consistently higher fecundity and body size than either of the founder parents up to the 20^th ^generation. If exotic genetically modified mosquitoes are introduced, it is, therefore, probable that the hybrids may live longer, exhibit higher fecundity, have larger body size and will likely feed more. Increased hybrid fitness would lead to stabilizing selection, rendering the refractory genes more difficult to be fixed. Given that greater mosquito density, fecundity, human biting habits and longevity of anopheline mosquito vectors are positively correlated with increased vectorial capacity [28,29], these results strongly indicate the need to release genetically modified mosquitoes that have the same or very similar genetic makeup to that of the native populations.

## Authors' contributions

DM and TG conducted fitness studies and prepared the manuscript. DZ, AP and GZ analysed the data and assisted with manuscript preparation. GY, LG, and JCB conceived the design of the study. All authors read and approved the final manuscript.
